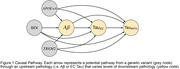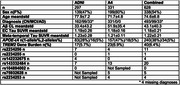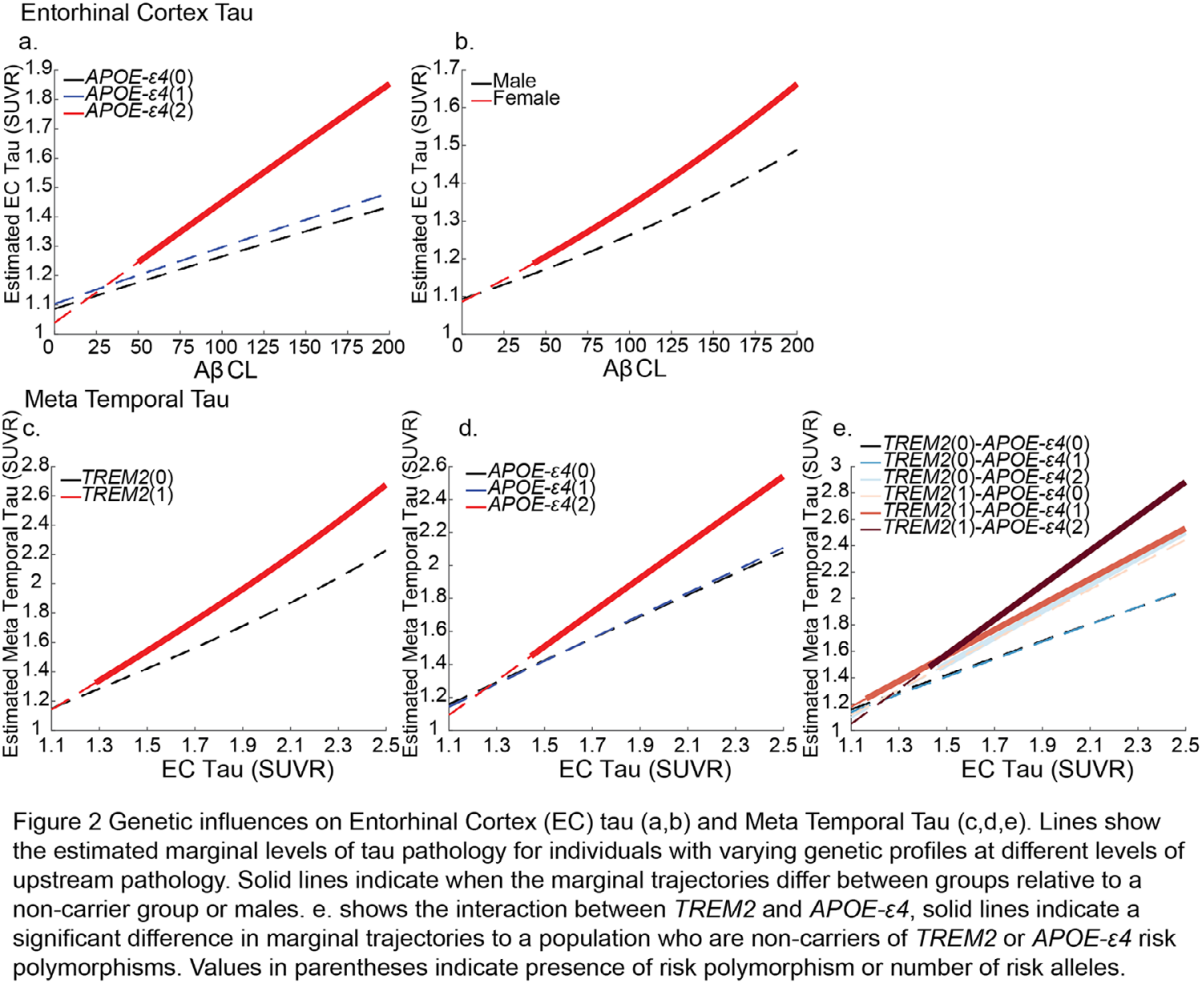# Genetic factors affect tau deposition in entorhinal cortex and neocortex

**DOI:** 10.1002/alz.084883

**Published:** 2025-01-03

**Authors:** Joseph Giorgio, Caroline Warly Solsberg, Yilin Wang, Jennifer S. Yokoyama, Jingshen Wang, William J. Jagust

**Affiliations:** ^1^ University of California, Berkeley, Berkeley, CA USA; ^2^ The University of Newcastle, Callaghan, NSW Australia; ^3^ University of California San Francisco, San Francisco, CA USA; ^4^ University of Iowa, Iowa City, IN USA; ^5^ Memory and Aging Center, UCSF Weill Institute for Neurosciences, University of California, San Francisco, San Francisco, CA USA; ^6^ University of California, San Francisco, San Francisco, CA USA; ^7^ Lawrence Berkeley National Laboratory, Berkeley, CA USA

## Abstract

**Background:**

The amyloid cascade hypothesis posits a sequence of events proceeding from amyloid‐β (Aβ) deposition to entorhinal cortical (EC) tau to neocortical (meta‐temporal) tau. This study examined how genetics may modify relationships between these variables on the AD pathway.

**Methods:**

We used causal path analyses to model effects of sex, *APOE*‐ε4 (0,1,2 alleles), and genetic risk for neuroinflammation on Aβ, EC tau and meta temporal tau. We modelled main effects and pairwise interactions between genetic profiles and earlier pathology on downstream pathologies, accounting for the full mediation path from Aβ→ EC tau → meta‐temporal tau (Figure 1.). Participants were 628 individuals from ADNI and A4 with whole genome sequencing, Aβ‐PET (Centiloids, CL) and [18F]flortaucipir (FTP)‐PET SUVR. Neuroinflammation gene burden (1/0) was defined as one or more rare polymorphisms on the coding region of the *TREM2* gene that have been previously associated with AD (Table 1.).

**Results:**

We observed interactions between Aβ and *APOE*‐ε4(2‐alleles) (β = 0.002, P = 0.002), and Aβ and sex(F) (β = 0.0008, p = 0.032) on EC tau: higher Aβ was associated with more EC tau in *APOE*‐ε4 homozygotes and females (Figure 2ab.). Next, we observed interactions between EC tau and *APOE*‐ε4(2‐alleles) (β = 0.364, P<0.001), and EC tau and *TREM2*(1) (β = 0.309, P<0.001) on the spread of tau to meta‐temporal regions: both *APOE*‐ε4 homozygotes and individuals with a *TREM2* risk allele had greater downstream meta‐temporal tau burden for a given level of EC tau (Figure 2cd). A significant interaction between *APOE*‐ε4 and *TREM2* (β = 0.0186, p = 0.0168) indicated highest risk in those with a *TREM2* risk allele and 1 or 2 ε4 alleles (Figure 2e.).

**Conclusion:**

Genetic factors affect tau pathological burden above and beyond the effects of Aβ. Both females and *APOE*‐ε4 homozygotes express tau deposition in the EC at lower Aβ CL than males and non‐homozygotes. Neocortical tau deposition is related to EC tau and is greater in *APOE*‐ε4 homozygotes and individuals with a genetic risk for impaired neuroinflammatory responses. These findings may explain variable effects of anti‐amyloid treatments and provide insights into the potential biological drivers of tau spread.